# Recent Advances in Plant–Insect Interactions

**DOI:** 10.3390/ijms241411338

**Published:** 2023-07-12

**Authors:** Francesca Barbero, Massimo E. Maffei

**Affiliations:** 1 Department of Life Sciences and Systems Biology, University of Turin, Via Accademia Albertina, 13-1023 Turin, Italy; francesca.barbero@unito.it; 2 Department of Life Sciences and Systems Biology, Plant Physiology Unit, University of Turin, Via Quarello 15/a, 10135 Turin, Italy

Plant–insect interaction is a fast-developing research field that continues to increase the interest of numerous scientists, many of whom come from heterogeneous backgrounds. This variety reflects the complexity and the multifaceted associations from which plants and insect interactions may originate.

Mutualism, pollination, and biotrophy boost the co-evolution of plant and insect responses. In the case of antagonistic associations, plants have evolved defenses against various insect feeding strategies, which are counterbalanced by the ability of insects to detoxify plant chemicals or to react specifically to plant compounds. The success of plants in withstanding insect herbivory depends on their ability to quickly recognize and decipher the incoming signal and adequately respond to a wide array of attacking herbivores. Whilst this topic has been widely investigated, less attention has been paid to how insects use plant signals to their own advantage to detect high-quality food, choose where to lay their eggs, or find their prey.

Current research on plant–insect interactions has mainly focused on genomics and proteomics, which are late events induced by biotic stress. Early events, within the first seconds to minutes, are responsible for the recognition and triggering of the signal transduction pathways, preceding both genomic and proteomic responses. For both plants and insects, sensing and communication are key features that can improve fitness and grant survival in contrasting environments.

Understanding how plants counteract herbivore aggression equals our understanding of insect herbivores’ defense against plant countermeasures. In this Special Issue, we explore the genomics and metabolomics of plant–insect interactions with a collection of 19 papers focusing on the effects of some of the most devastating herbivores on their host plants, including planthoppers, thrips, and aphids, among others.

## 1. Overview of Plant–Insect Interaction

Gen-ichiro Arimura and co-workers focus on the nature of the transcriptional regulation of the jasmonic acid (JA)-responsive plant defensin gene *PDF1.2* and abscisic acid (ABA)-responsive UDP-D-glucuronate 4-epimerase 6 (*GEA6*) in *Arabidopsis thaliana* [1]. This paper introduces transcriptional reprogramming in the defense response against herbivores in higher plants, which is further and deeply elaborated by the review paper of Biao Jin and co-workers [2]. The review summarizes plants’ interactions with herbivores from early events involved in the perception of physical and chemical stimuli—involving both membrane potential variations and the triggering of secondary messengers such as Ca^2+^ and H_2_O_2_—to the activation of phytohormone pathways and the attraction of herbivores’ predators through the emission of herbivore-induced volatile organic compounds (HIVOCs). This review introduces several aspects that are deeply explored in the papers comprising this Special Issue.

## 2. Planthopper–Rice Interactions

The paper by Guangcun He and co-workers on the interaction of the brown planthopper (*Nilaparvata lugens*, BPH) with resistant and susceptible rice varieties reveals differentially accumulated metabolites (DAMs) and expressed genes (DEGs) with flavonoids and salicylic acid and genes related to stimulus, with them being upregulated in the resistant variety [3]. They provide compelling evidence that transcriptomic and metabolic information is important for understanding rice–BPH interactions. 

BPH stress resilience is the subject of a paper by Ayushi Gupta and Suresh Nair [4], who studied BPH DNA methylation patterns under pesticide and nutritional stress across BPH life stages. Their results show that DNA methylation in BPH is insect life stage specific and varies with environmental cues. Furthermore, the disruption of the BPH methylome influences gene expression, affecting BPH stress resilience.

In the white-backed planthopper (WBPH), *Sogatella furcifera*, Maolin Hou and co-workers evaluated the salivary gland expression of the mucin-like salivary protein (SFMLP) by using both double-stranded RNA SFMLP (dsSFMLP) and SFMLP gene knockdown [5]. Their results showed that SFMLP has a function in rice defense and is essential for feeding in WBPH, as the salivary protein is involved in the formation of the salivary sheath.

## 3. Aphid–Plant Interactions

Aphids, with particular reference to the generalist *Myzus persicae*, show high resistance to pesticides, which has prompted the search for new environmentally-friendly bioactive molecules to control this pest. Francisca Blanco-Herrera and co-workers showed that pectin-derived oligogalacturonides (OGs) improve Arabidopsis’ performance against *M. persicae* by identifying defense mechanisms involving the production of callose and ROS and the expression of genes related to the salicylic acid (SA) signaling pathway [6]. The treatment of Arabidopsis with OGs successfully increased resistance to the aforementioned aphid. 

Marilyne Uzest and co-workers studied the acrostyle, an aphid organ that produces stylin proteins able to bind to plant viruses transiently. They found that a salivary effector (Mp10), which aphids inject into the plant’s cytoplasm during feeding, can bind the acrostyle by probably interacting with stylin-03 of both *M. persicae* and *Acyrthosiphon pisum* [7]. By using RNA interference (RNAi) of both stylin-03 and stylin-01, the authors showed that the acrostyle plays a role in delivering effectors produced by the aphids into the plant cell cytosol.

*A. pisum’s* feeding activity has been studied through its interaction with wild-type pea in the context of climate change, with particular reference to elevated CO_2_ (eCO_2_) levels. Christine H. Foyer and co-workers showed that the phytohormones strigolactone (SL) and gibberellin are involved in host susceptibility in pea–*A. pisum* interaction, but the aphid–plant compatibility control of these phytohormones is not changed by eCO_2_ [8].

## 4. *Spodoptera*–Plant Interactions

Plant interactions with pollinators imply the perception of insects, the induction of early signals and responses, short- and long-distance signal transduction, phenotypic changes, and the ability of the insect to co-evolve intricate patterns of associations with plants. Kongming Wu and co-workers reported on the ability of the moth *Spodoptera exigua* to visit different plant species and analyzed the pollen attached to migratory individuals, which at the larval stage are major crop pests [9]. The authors detected a high level of conspecific attraction, identifying patterns of migration and interactions between the moths and their host plants which allows for the design of strategies to preserve and optimize ecosystem services.

The resistance of Arabidopsis to *S. littoralis* herbivory has been studied by focusing on CALMODULIN1 (CAM1) and WRKY53 function. Yingbai Shen and co-workers [10] confirmed that CAM1 interacts with the transcription factor WRKY53, which in turn binds to the promoter regions of lipoxygenases 3 (*LOX3*) and lipoxygenases 4 (*LOX4*) by negatively regulating their gene expression. Eventually, the plant resistance to herbivory is negatively regulated by CAM1 and WRKY53 through regulation of the JA biosynthesis pathway. These results underline the role of WRKY53 in plant resistance to insects.

## 5. *Helicoverpa armigera*–Plant Interactions

*Helicoverpa armigera* (namely the pod borer) is known for being one of the major species responsible for crop losses. Rohini Sreevathsa and co-workers analyzed the flavonoid biosynthesis in one of the pigeonpea wild relatives, *Cajanus platycarpus*, by focusing their study on flavonoid 3′5′ hydroxylase (CpF3′5′H_2), an enzyme that represents an important branch-point in the production of *C. platycarpus* flavonoids [11]. By using CpF3′5′H_2-overexpressed transgenic tobacco lines, the authors demonstrated that increasing flavonoids, polyphenols, and ROS scavenging ability is a successful strategy in the management of *H. armigera* biotic stress.

Marcio C. Silva-Filho and co-workers investigated the role of soybean (*Glycine max*) peptidase inhibitors (SPIs) in the resistance mechanisms of *H. armigera* larvae [12]. They found that the adaptation of *H. armigera* to soybean SPIs involves upregulation in the digestive system of insect genes encoding for serine peptidases. The epigenetics of SPI adaptation were studied by methylome analysis, showing that DNA methylation plays a regulatory role in insect adaptation to plant anti-herbivore defense proteins with transcriptional reprogramming that can be transmitted across generations.

## 6. Plant Interactions with Thrips

Thrips are known for their high fecundity, which leads to a significant increase in thrips’ resistance to pesticides. The western flower thrip, *Frankliniella occidentalis*, is a serious, worldwide invasive pest. Junrui Zhi and co-workers reported the glutathione S-transferase *FoGSTd1* and *FoGSTs1* gene expression of *F. occidentalis* after feeding on exogenous JA- and methyl JA-induced kidney bean plants [13]. The RNAi downregulation of *FoGSTd1* and *FoGSTs1* has been found to reduce the anti-adaptive ability of *F. occidentalis* to JA- or MeJA-induced defenses in kidney bean plants.

Enrique Ibarra-Laclette and co-workers used metabolomics and transcriptomics approaches to investigate the mechanisms exploited by *Persea americana*, cv. Hass (Hass avocados) to detect and react to the Mexican fruit fly, *Anastrepha ludens* [14]. Transcriptomic analysis revealed the presence of several DEGs and the induction of hypersensitive response (HR) upon oviposition, with the production of phytoalexins and ROS and the induction of chitin receptors. Metabolomic analysis revealed the production of polyphenols with flavonoid biosynthetic pathways involved in the response to oviposition. The finding of potential ovicide metabolites suggests that these molecules could be useful as a natural defense against herbivore attack.

## 7. Interactions of Plants with the Diamondback Moth (DBM), *Plutella xylostella*

*Plutella xylostella* is one of the most destructive pests for cruciferous vegetables, with the species having a wide distribution and the ability to adapt quickly to new environments. Shijun You and co-workers analyzed the insect trehalose transporter (*PxTret1-like*) and reported that the expression pattern of this gene is affected by the ambient temperature [15]. By using a CRISPR/Cas9 system, they obtained the knockout mutation of *PxTret1-like* and showed that the gene regulates the main sugar—trehalose—content in the *P. xylostella’s* body by affecting the insect’s ability to adapt to temperature, develop, and reproduce.

The emission of VOCs is one of the typical responses of plants to herbivory. By using confocal microscopy and non-invasive micro-test technology, Yingbai Shen and co-workers demonstrated that the volatile linalool, whose emission typically improves plant resistance to *P. xylostella*, increases ROS (H_2_O_2_) production and triggers calcium signaling that involves interactions with calmodulins (CAM3 and CAM7) [16]. Moreover, linalool enhances plant defenses by modulating the expression of JA-related and other defense genes.

## 8. Plant–Insect Interactions via Volatile Compounds

VOCs are also effective chemical signals in plant–insect interactions. Sulfur volatiles released from guava (*Psidium guajava*) reduce infestation of the Asian citrus psyllid (ACP, *Diaphorina citri*). Xinnian Zeng and co-workers report on the defense responses of sweet orange (*Citrus sinensis*) and show that sulfur volatiles emitted by guava play a role in plant–plant communications and trigger anti-herbivore activities against ACP [17]. Dimethyl disulfide (DMDS) emitted by guava suppresses ACP performance and additionally activates defense responses in neighboring plants, indicating that DMDS could be used as a strategy to manage ACP in citrus orchards.

The rubber bark beetle *Euplatypus parallelus* is widely diffused in rubber-planting areas and is attracted by semiochemicals emitted by rubber trees. Jixing Guo and co-workers studied the role of *E. parallelus* odorant-binding protein 2 (EparOBP2) in the recognition process of semiochemicals by cloning the cDNA sequence of *EparOBP2* [18]. Fluorescence competitive binding assays confirmed that EparOBP2 has broad binding properties toward nine semiochemicals, including α-pinene and myrcene. Therefore, EparOBP2 may be involved in semiochemical chemoreception and is a potential target for the integrated management of *E. parallelus*.

## 9. Plant–Insect–Bacteria Interactions

Bacteria are commonly associated with insect hosts, and some bacterial symbionts, such as *Wolbachia,* have evolved mutualistic interactions. The fly *Eurosta solidaginis* induces galls in the apical meristems of *Solidago altissima* and hosts a strain of *Wolbachia*. Edward F. Connor and co-workers investigated the potential role of a bacterial symbiont in gall induction by sequencing the metagenome of *E. solidaginis* and its *Wolbachia* symbiont *w*Esol [19]. Annotation of the *w*Esol genome may reveal whether genes involved in phytohormone pathways are present in *w*Esol and can potentially identify effector proteins secreted by *w*Esol. The authors showed that *w*Esol is absent from the salivary glands of *E. solidaginis*, suggesting that *w*Esol does not contribute to gall induction by its host.

## 10. Editors’ Conclusions and Future Perspectives

The papers published in this Special Issue provide novel insights into the complex dynamics of plant–insect interactions, shedding light on the intricate molecular mechanisms that shape their co-evolutionary relationships. This knowledge has far-reaching implications for various fields, including agriculture, ecology, and pest management.

Still, several aspects of such a vital interaction between plants and insects remain to be clarified. Further exploration of the overall plant signaling pathways and their crosstalk with insect-derived molecules should be advocated. Understanding the intricacies of these signaling networks will provide valuable insights into the communication between plants and insects and may reveal potential targets for manipulation to control plant or insect resistance.

The latest high-throughput sequencing and silencing technologies to study plant–insect interactions can uncover previously unknown molecular players and elucidate complex regulatory networks. Integrating genomic, transcriptomic, and proteomic approaches will provide a comprehensive understanding of the molecular basis of these interactions. In addition, the emergence of new tools such as CRISPR-Cas9 genome editing technology offers exciting opportunities for the targeted manipulation of both plants and insects.

By bringing together experts from molecular biology, ecology, entomology, and agronomy, it would be possible to foster innovative research approaches that enable a holistic understanding of plant–insect interactions and pave the way for developing sustainable strategies for crop protection, ecosystem services, and environmental conservation.

## References

[1] Kawaguchi J., Hayashi K., Desaki Y., Ramadan A., Nozawa A., Nemoto K., Sawasaki T., Arimura G.-I. (2023). JUL1, Ring-Type E3 Ubiquitin Ligase, Is Involved in Transcriptional Reprogramming for ERF15-Mediated Gene Regulation. Int. J. Mol. Sci..

[2] Mostafa S., Wang Y., Zeng W., Jin B. (2022). Plant Responses to Herbivory, Wounding, and Infection. Int. J. Mol. Sci..

[3] Zhang Q., Li T., Gao M., Ye M., Lin M., Wu D., Guo J., Guan W., Wang J., Yang K. (2022). Transcriptome and Metabolome Profiling Reveal the Resistance Mechanisms of Rice against Brown Planthopper. Int. J. Mol. Sci..

[4] Gupta A., Nair S. (2022). Heritable Epigenomic Modifications Influence Stress Resilience and Rapid Adaptations in the Brown Planthopper (*Nilaparvata lugens*). Int. J. Mol. Sci..

[5] Liu Y., Yi J., Jia H., Miao Y., Hou M. (2022). *Sogatella furcifera* Saliva Mucin-like Protein Is Required for Feeding and Induces Rice Defences. Int. J. Mol. Sci..

[6] Silva-Sanzana C., Zavala D., Moraga F., Herrera-Vásquez A., Blanco-Herrera F. (2022). Oligogalacturonides Enhance Resistance against Aphids through Pattern-Triggered Immunity and Activation of Salicylic Acid Signaling. Int. J. Mol. Sci..

[7] Deshoux M., Monsion B., Pichon E., Jiménez J., Moreno A., Cayrol B., Thébaud G., Mugford S.T., Hogenhout S.A., Blanc S. (2022). Role of Acrostyle Cuticular Proteins in the Retention of an Aphid Salivary Effector. Int. J. Mol. Sci..

[8] Swiegers H.W., Karpinska B., Hu Y., Dodd I.C., Botha A.-M., Foyer C.H. (2022). The Effects of High CO_2_ and Strigolactones on Shoot Branching and Aphid-Plant Compatibility Control in Pea. Int. J. Mol. Sci..

[9] Jia H., Wang T., Li X., Zhao S., Guo J., Liu D., Liu Y., Wu K. (2023). Pollen Molecular Identification from a Long-Distance Migratory Insect, *Spodoptera exigua*, as Evidenced for Its Regional Pollination in Eastern Asia. Int. J. Mol. Sci..

[10] Jiao C., Li K., Zuo Y., Gong J., Guo Z., Shen Y. (2022). CALMODULIN1 and WRKY53 Function in Plant Defense by Negatively Regulating the Jasmonic Acid Biosynthesis Pathway in Arabidopsis. Int. J. Mol. Sci..

[11] Tyagi S., Rathinam M., Dokka N., Chaudhary N., Satish L., Dash P.K., Shasany A.K., Sreevathsa R. (2023). *Cajanus platycarpus* Flavonoid 3′5′ Hydroxylase (CpF3′5′H_2) Confers Resistance to *Helicoverpa armigera* by Modulating Total Polyphenols and Flavonoids in Transgenic Tobacco. Int. J. Mol. Sci..

[12] Velasquez-Vasconez P.A., Hunt B.J., Dias R.O., Souza T.P., Bass C., Silva-Filho M.C. (2022). Adaptation of *Helicoverpa armigera* to Soybean Peptidase Inhibitors Is Associated with the Transgenerational Upregulation of Serine Peptidases. Int. J. Mol. Sci..

[13] Zhang T., Liu L., Jia Y., Zhi J., Yue W., Li D., Zeng G. (2022). Induced Resistance Combined with RNA Interference Attenuates the Counteradaptation of the Western Flower Thrips. Int. J. Mol. Sci..

[14] Aluja M., Vázquez-Rosas-Landa M., Cerqueda-García D., Monribot-Villanueva J.L., Altúzar-Molina A., Ramírez-Vázquez M., Velázquez-López O., Rosas-Saito G., Alonso-Sánchez A.G., Ortega-Casas R. (2023). Assessment of the Molecular Responses of an Ancient Angiosperm against Atypical Insect Oviposition: The Case of Hass Avocados and the Tephritid Fly *Anastrepha ludens*. Int. J. Mol. Sci..

[15] Zhou H., Lei G., Chen Y., You M., You S. (2022). PxTret1-like Affects the Temperature Adaptability of a Cosmopolitan Pest by Altering Trehalose Tissue Distribution. Int. J. Mol. Sci..

[16] Jiao C., Gong J., Guo Z., Li S., Zuo Y., Shen Y. (2022). Linalool Activates Oxidative and Calcium Burst and CAM3-ACA8 Participates in Calcium Recovery in Arabidopsis Leaves. Int. J. Mol. Sci..

[17] Ling S., Qiu H., Xu J., Gu Y., Yu J., Wang W., Liu J., Zeng X. (2022). Volatile Dimethyl Disulfide from Guava Plants Regulate Developmental Performance of Asian Citrus Psyllid through Activation of Defense Responses in Neighboring Orange Plants. Int. J. Mol. Sci..

[18] Cui G., Zhou X., Wang Q., Zhang K., Qin L., Guo J. (2023). The Sequence Characteristics and Binding Properties of the Odorant-Binding Protein 2 of *Euplatypus parallelus* to Semiochemicals. Int. J. Mol. Sci..

[19] Fiutek N., Couger M.B., Pirro S., Roy S.W., de la Torre J.R., Connor E.F. (2023). Genomic Assessment of the Contribution of the *Wolbachia* Endosymbiont of *Eurosta solidaginis* to Gall Induction. Int. J. Mol. Sci..

